# sTREM-1 predicts mortality in hospitalized patients with infection in a tropical, middle-income country

**DOI:** 10.1186/s12916-020-01627-5

**Published:** 2020-07-01

**Authors:** Shelton W. Wright, Lara Lovelace-Macon, Viriya Hantrakun, Kristina E. Rudd, Prapit Teparrukkul, Susanna Kosamo, W. Conrad Liles, Direk Limmathurotsakul, T. Eoin West

**Affiliations:** 1 Division of Pediatric Critical Care Medicine, Department of Pediatrics, University of Washington, Seattle, WA 98104 USA; 2 Division of Pulmonary, Critical Care and Sleep Medicine, Department of Medicine, University of Washington, Seattle, WA 98195 USA; 3 Mahidol-Oxford Tropical Medicine Research Unit, Faculty of Tropical Medicine, Mahidol University, Bangkok, 10400 Thailand; 4 Department of Critical Care Medicine, University of Pittsburgh, Pittsburgh, PA 15213 USA; 5 Department of Internal Medicine, Sunpasitthiprasong Hospital, Ubon Ratchathani, 34000 Thailand; 6 Division of Allergy and Infectious Diseases, Department of Medicine, University of Washington, Seattle, WA 98195 USA; 7 Department of Tropical Hygiene, Faculty of Tropical Medicine, Mahidol University, Bangkok, 10400 Thailand; 8 University of Washington, Box 359640, 325 Ninth Ave., Seattle, WA 98104 USA

**Keywords:** Sepsis, Soluble triggering receptor expressed by myeloid cells 1, sTREM-1, Low- and middle-income countries, LMIC

## Abstract

**Background:**

Few studies of biomarkers as predictors of outcome in infection have been performed in tropical, low- and middle-income countries where the burden of sepsis is highest. We evaluated whether selected biomarkers could predict 28-day mortality in infected patients in rural Thailand.

**Methods:**

Four thousand nine hundred eighty-nine adult patients admitted with suspected infection to a referral hospital in northeast Thailand were prospectively enrolled within 24 h of admission. In a secondary analysis of 760 patients, interleukin-8 (IL-8), soluble tumor necrosis factor receptor 1 (sTNFR-1), angiopoietin-1 (Ang-1), angiopoietin-2 (Ang-2), and soluble triggering receptor expressed by myeloid cells 1 (sTREM-1) were measured in the plasma. Association with 28-day mortality was evaluated using regression; a parsimonious biomarker model was selected using the least absolute shrinkage and selection operator (LASSO) method. Discrimination of mortality was assessed by receiver operating characteristic curve analysis and verified by multiple methods.

**Results:**

IL-8, sTNFR-1, Ang-2, and sTREM-1 concentrations were strongly associated with death. LASSO identified a three-biomarker model of sTREM-1, Ang-2, and IL-8, but sTREM-1 alone provided comparable mortality discrimination (*p* = 0.07). sTREM-1 alone was comparable to a model of clinical variables (area under receiver operating characteristic curve [AUC] 0.81, 95% confidence interval [CI] 0.77–0.85 vs AUC 0.79, 95% CI 0.74–0.84; *p* = 0.43). The combination of sTREM-1 and clinical variables yielded greater mortality discrimination than clinical variables alone (AUC 0.83, 95% CI 0.79–0.87; *p* = 0.004).

**Conclusions:**

sTREM-1 predicts mortality from infection in a tropical, middle-income country comparably to a model derived from clinical variables and, when combined with clinical variables, can further augment mortality prediction.

**Trial registration:**

The Ubon-sepsis study was registered on ClinicalTrials.gov (NCT02217592), 2014.

## Background

Sepsis, defined as organ dysfunction from a dysregulated immune response to infection, is a feared complication of infection and a major cause of death worldwide. Low- and middle-income countries (LMICs)—where etiologies, host factors, and clinical management may differ from high-income settings—are particularly impacted [1, 2]. Sepsis-related mortality in tropical Southeast Asia is especially high and related to a heterogenous group of causes [2, 3].

Early and accurate assessment of the sepsis-related clinical trajectory is imperative, especially in settings with developing emergency transportation infrastructure and limited tertiary healthcare centers [1]. Identifying patients at the lowest and highest risk of deterioration permits accurate triage and judicious use of scarce resources. Thailand is an upper middle-income country with a tropical climate [4]. The northeast region of Thailand has diverse causes of sepsis, including leptospirosis, melioidosis, dengue, scrub and murine typhus, and malaria [3, 5–7]. In one referral hospital in this region, where some critical care resources are limited, we have reported that septic patients with respiratory failure and shock are commonly managed on general medical wards [8]. Optimizing prediction of outcomes from sepsis in environments such as this is essential.

Clinical scoring systems, such as the Sequential Organ Failure Assessment (SOFA) score, have been proposed as methods for predicting sepsis-related outcomes, including mortality [9]. Furthermore, modified versions of the SOFA score have been successful in predicting outcomes in LMICs, including parts of Southeast Asia [10, 11]. Clinical scoring systems, including SOFA, are frequently based on gradations of advanced cardiorespiratory support. However, in many LMICs, critically ill patients may be treated outside traditional intensive care units or without advanced support capabilities, potentially making the application of such clinical scores challenging [10].

Biomarkers, particularly those measurable in the peripheral venous blood, have become increasingly popular as complementary or alternative methods for predicting sepsis-related outcomes. Inflammatory markers such as procalcitonin and C-reactive protein have been proposed as both diagnostic and prognostic biomarkers in sepsis, although their performance in tropical Southeast Asia is inconsistent [12, 13]. Other inflammatory markers such as soluble triggering receptor expressed by myeloid cells 1 (sTREM-1), interleukin-8 (IL-8), and soluble tumor necrosis factor receptor 1 (sTNFR-1) have shown promise, mostly in high-resource settings with temperate climates, in predicting sepsis-related mortality [14, 15]. Similarly, biomarkers of endothelial quiescence and activation, including angiopoietin-1 (Ang-1) and angiopoietin-2 (Ang-2), have also been reported to be accurate predictors of sepsis outcome [16]. However, the external validity of the performance of these biomarkers in predicting sepsis outcomes in under-resourced tropical countries—where the etiologic pathogens, host characteristics, and clinical management strategies may be distinct—is not established [1].

In this study, we reviewed the existing literature to identify five candidate biomarkers with compelling predictive evidence in populations with sepsis. We tested the hypothesis that these biomarkers could predict 28-day mortality in adults hospitalized with infection of various etiologies and at risk for sepsis in a tropical setting in rural Thailand.

## Methods

### Study design, setting, and participants

Subjects aged 18 years or older admitted to Sunpasitthiprasong Hospital in Ubon Rachathani, Thailand, with suspected infection were prospectively enrolled between 2013 and 2017 and have been reported previously [7, 10, 11]. Enrollment prospectively occurred within 24 h of admission to the study hospital if subjects possessed at least three documented systemic manifestations of infection, as proposed by the 2012 Surviving Sepsis Campaign [17]. Plasma samples were obtained at the time of enrollment. As Sunpasitthiprasong Hospital is a referral center, many patients are transferred from other hospitals in the region, and these patients differ from patients who present to the study hospital emergency department (ED) by clinical characteristics and outcome [11]. Transfer from another facility was therefore considered as a possible effect modifier of the relationship between biomarker and outcome, and transfer status was included as an interaction term in the regression models. Sample size estimates were based on the presumption of an interaction and the need for stratification of the cohort by transfer status. Assumptions were based on the published area under the receiver operating curve (AUC) for mortality discrimination by SOFA score and on the distribution of plasma IL-8 concentrations in a cohort of melioidosis patients from northeast Thailand [14, 18]. Analysis of 380 patients in each stratum would yield 99% power to detect improvement in AUC relative to an AUC of 0.75, assuming 10% non-survivors, log mean cytokine (variance) of 1.5 (0.3) in non-survivors, log mean cytokine (variance) of 1.0 (0.3) in survivors, and alpha 0.05 [19]. Three hundred eighty patients were selected by random sampling from the 551 patients presenting initially to the Sunpasitthiprasong ED, and 380 patients were randomly selected from the 3240 individuals transferred to the study hospital within 24 h of initial presentation. The plasma was available, and 28-day mortality outcome was known, for all 760 subjects selected.

### Clinical definitions

A modified Sequential Organ Failure Assessment (modified SOFA) score was calculated for all subjects at the time of enrollment, given the absence of some data points such as inotrope and vasopressor agent doses and partial pressure of oxygen in arterial blood (PaO_2_). This modified SOFA score has been previously described [11]. For the cardiovascular component of the SOFA score, 2 out of 4 points were given for receipt of dobutamine or dopamine and 3 out of 4 points were given for receipt of epinephrine or norepinephrine. For the respiratory component of the SOFA score, 2 out of 4 points were given if advanced respiratory support (endotracheal tube or mechanical ventilation) was utilized but arterial blood gas results were not available.

### Biomarker assays

Plasma concentrations of IL-8, sTNFR-1, Ang-1, Ang-2, and sTREM-1 were measured using an electrochemiluminescence multiplex assay (Meso Scale Discovery, Rockville, MD), as previously described [20]. Samples were diluted due to the sensitivity of the assays, and upper and lower limits of detection were determined by the manufacturer’s software.

### Biomarker selection, model development, and analysis

A literature review was performed of biomarkers as predictors of mortality in sepsis. Five biomarkers (IL-8, sTNFR-1, Ang-1, Ang-2, and sTREM-1) were identified as having strong mortality discrimination in diverse populations with sepsis, primarily in high-resource settings [21–25]. After biomarker quantification, an Ang-2:Ang-1 ratio was calculated. Differences in biomarker concentrations between the two groups were evaluated using the Mann-Whitney *U* test. Biomarkers were log_10_-transformed, and associations with 28-day mortality were evaluated using logistic regression. Prior to further analyses, tests of interaction were performed to evaluate the change in biomarker prediction of mortality by transfer status. Candidate models of mortality prediction were initially developed using pre-specified clinical variables including age, sex, Charlson Comorbidity Index, transfer status, a modified SOFA score, and the individual biomarker analytes. All models were assessed for the goodness of fit using the Hosmer-Lemeshow chi-square analysis. Receiver operating characteristic curve analysis was performed to evaluate mortality discrimination. To simplify the prediction model to the fewest number of variables possible, all five analytes and Ang-2:Ang-1 were subjected to logistic regression analysis by the least absolute shrinkage and selection operator (LASSO) methodology in which lambda was selected by the Akaike Information Criterion; the selected biomarkers were confirmed using the largest lambda within one standard error of the minimal mean squared prediction error based on 10-fold internal cross-validation [26, 27].

The LASSO-selected analytes and clinical variables were evaluated as predictors of 28-day mortality by creating logistic regression models and comparing AUCs to further refine the model. Subsequently, several methods were employed to verify discrimination. (1) Optimism-corrected AUCs were generated by an internal validation of 1000 replication sets by bootstrapping [28]. (2) Discrimination ability was further assessed using integrated discrimination improvement analysis (IDI) [29, 30]. (3) Nested models were subsequently compared using the likelihood ratio (LR) test [31]. (4) Finally, the net benefit of the combined biomarker and clinical variable model over the clinical variable model alone was assessed using a decision curve analysis [32]. Analyses were performed using Stata/SE version 14.2 (College Station, TX). Two-sided *p* values < 0.05 were considered statistically significant.

## Results

### Clinical characteristics

The clinical characteristics of the sampled cohort are shown in Table 1. The median age was 59 years (interquartile range (IQR) 41–73), and the median Charlson Comorbidity Index score was 2 (IQR 0–4). On enrollment, the median modified SOFA score was 3 (IQR 1–5). The 28-day mortality rate was 14%. The clinical characteristics of the patients stratified by initial ED presentation or transfer from another facility can be seen in see Additional file 1.

**Table 1:** Patient characteristics

Characteristics	Cohort (*n* = 760)
Demographics	
Age in years, median (IQR)	59 (41–73)
Male sex, *N* (%)	401 (53)
Pre-existing conditions	
Charlson Comorbidity Index, median (IQR)	2 (0–4)
Diabetes, *N* (%)	157 (21)
Chronic liver disease, *N* (%)	18 (2)
Chronic kidney disease, *N* (%)	85 (11)
Chronic cardiovascular disease, *N* (%)	47 (6)
Chronic lung disease, *N* (%)	60 (8)
Cancer, *N* (%)	17 (2)
HIV, *N* (%)	9 (1)
Modified SOFA score, median (IQR)	3 (1–5)
Subjects with modified SOFA score ≥ 2, *N* (%)	492 (65)
Died within 28 days, *N* (%)	110 (14)

### Biomarker concentrations in survivors and non-survivors

IL-8, sTNFR-1, Ang-1, Ang-2, and sTREM-1 concentrations measured in enrollment plasma samples were compared in survivors and non-survivors to 28-days (Table 2). Concentrations of IL-8, sTNFR-1, Ang-2, and sTREM-1, and the Ang-2:Ang-1 ratio were significantly lower in survivors compared to non-survivors (all *p* < 0.001). There was no difference in concentrations of Ang-1.

**Table 2:** Plasma biomarker concentration in survivors and non-survivors of infection

Biomarker (pg/ml)	All, median (IQR) (*n* = 760)^a^	Survivors, median (IQR) (*n* = 650)	Non-survivors, median (IQR) (*n* = 110)	*p* value^b^
IL-8	14 (7–55)	12 (6–31)	76 (31–933)	< 0.001
sTNFR-1	7749 (3650–17,354)	6491 (3408–14,346)	20,571 (11890–30,923)	< 0.001
Ang-1	2012 (925–3874)	1985 (899–3835)	2083 (989–4028)	0.58
Ang-2	8859 (4436–17,496)	7751 (4043–15,042)	23,939 (10432–42,212)	< 0.001
Ang-2:Ang-1	5 (2–16)	4 (2–14)	14 (4–35)	< 0.001
sTREM-1	405 (241–830)	361 (224–655)	1037 (647–1727)	< 0.001

^a^For Ang-1 and Ang-2, concentrations were measured in 748 samples

^b^*p* value calculated using the Mann-Whitney *U* test

### Association of biomarkers with death

The association between log_10_-transformed biomarker concentration and death was analyzed using logistic regression. Given the possibility of effect modification by transfer status, an interaction between each biomarker and transfer status on the association with death was first assessed in the models (see Additional file 2). A significant interaction with transfer status was found only for Ang-2 (*p* = 0.03). In the full cohort, increased concentrations of IL-8, sTNFR-1, sTREM-1, and Ang-2:Ang-1 each were significantly associated with death (all *p* < 0.001; Table 3). The association of Ang-2 with death was separately evaluated in the set of non-transferred patients and in the set of transferred patients; increased concentrations of this biomarker were significantly associated with death in both sets (*p* < 0.001 in both sets; see Additional file 3). However, in models adjusted for age, sex, Charlson Comorbidity Index, and modified SOFA score, no significant interaction of any biomarker with transfer status was found (see Additional file 2). In the full cohort, IL-8, sTNFR-1, Ang-2, sTREM-1, and Ang-2:Ang-1 remained associated with death when the models were adjusted for age, sex, transfer status, Charlson Comorbidity Index, and modified SOFA score at enrollment (all *p* < 0.001, Table 3). The biomarker with the highest point estimate for the odds of death was sTREM-1 (adjusted odds ratio (OR) 11.2 for each log_10_ increase in sTREM1, 95% confidence interval (CI) 4.9–25.9). All models had appropriate goodness of fit as assessed by the Hosmer-Lemeshow method (*p* > 0.05).

**Table 3:** Association of biomarkers with death at 28 days

Biomarker^a^	Unadjusted		Modified SOFA-adjusted^b^	
	OR (95% CI)	*p* value	OR (95% CI)	*p* value
IL-8	2.3 (1.9–2.9)	< 0.001	1.9 (1.5–2.4)	< 0.001
sTNFR-1	21.3 (10.6–42.8)	< 0.001	5.2 (2.3–12.1)	< 0.001
Ang-1	1.1 (0.8–1.6)	0.48	1.2 (0.8–1.8)	0.4
Ang-2	11.3 (6.6–19.3)	< 0.001	5.4 (2.8–10.3)	< 0.001
Ang-2:Ang-1	2.2 (1.7–3.0)	< 0.001	1.5 (1.1–2.2)	0.02
sTREM-1	33.0 (16.3–67.0)	< 0.001	11.2 (4.9–25.9)	< 0.001

^a^Biomarkers were log_10_-transformed before regression; each biomarker was assessed in separate models

^b^Models were adjusted for age, sex, transfer status, Charlson Comorbidity Index, and modified SOFA score

### Discrimination of mortality

**Fig. 1:**
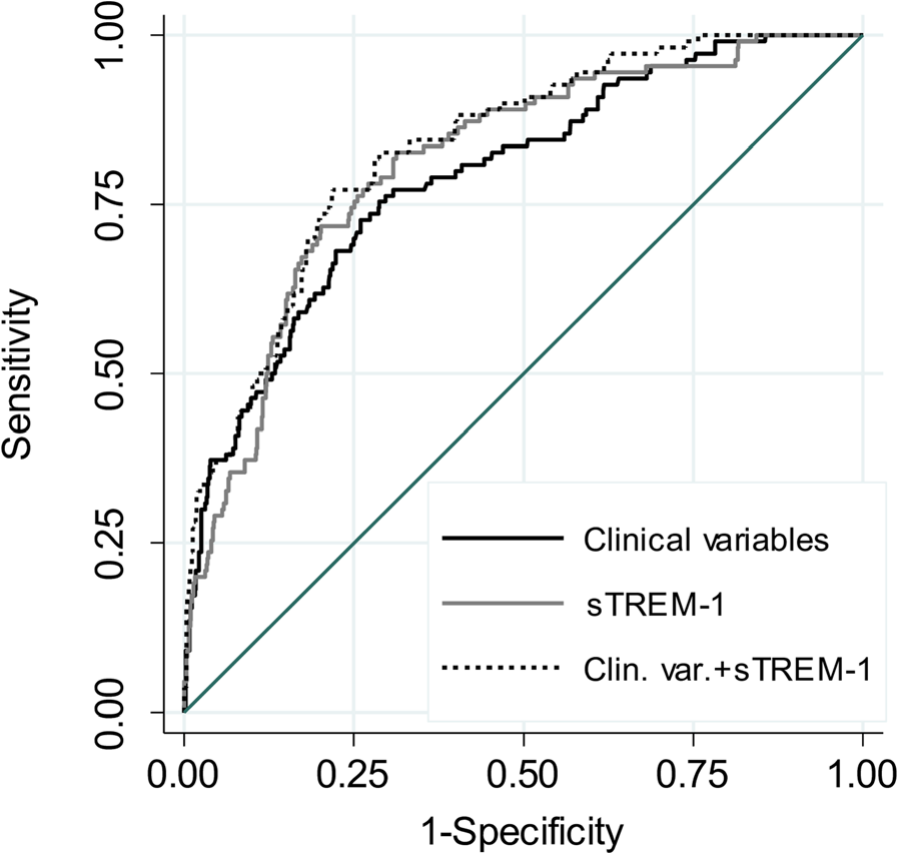
Discrimination of death by sTREM-1 and clinical variable models. Area under the receiver operating characteristic curve (AUC) for the clinical variable model including age, sex, Charlson Comorbidity Index, transfer status, and modified SOFA score; an sTREM-1 model (sTREM-1); and a model combining the clinical variable model and sTREM-1 (Clin. var. + sTREM-1) for 28-day mortality discrimination

In order to develop the most parsimonious biomarker model to predict mortality, LASSO regression was performed on the five analytes and on the Ang-2:Ang-1 ratio. This procedure selected sTREM-1, Ang-2, and IL-8 (see Additional file 7). Discrimination of mortality by the LASSO-selected three-biomarker model was determined by calculating the area under the receiver operating characteristic curve (AUC) 0.83, 95% CI 0.79–0.87. Compared to each of the biomarkers alone (Table 4), the three-biomarker model was superior to both Ang-2 and IL-8 but was not significantly better than sTREM-1 (AUC 0.81, 95% CI 0.77–0.85, *p* = 0.07), suggesting that sTREM-1 alone may provide comparable mortality discrimination. To evaluate how sTREM-1 performed relative to the available clinical data, a clinical variable model—comprising age, sex, Charlson Comorbidity Index, transfer status, and modified SOFA score—was developed. sTREM-1 had comparable mortality discrimination as the clinical variable model (AUC 0.81, 95% CI 0.77–0.85 vs AUC 0.79, 95% CI 0.74–0.84; *p* = 0.43; Table 5, Fig. 1). To determine whether sTREM-1 augmented clinical data, sTREM-1 was added to the clinical variable model. For the combined model, mortality discrimination (AUC 0.83, 95% CI 0.79–0.87) was significantly greater than for the clinical variable model alone (*p* = 0.004) and was borderline better than sTREM-1 alone (*p* = 0.05). Several other methods were used to verify these observations. The sTREM-1 models and the clinical variable model demonstrated minimal bias in optimism-adjusted AUCs after bootstrap validation (see Additional file 4). Discrimination analysis by IDI and the LR test showed significant improvement of the clinical variable model when sTREM-1 was added (IDI: *p* < 0.001, see Additional file 4; LR = 1.6 × 10^−9^, see Additional file 5). Finally, decision curve analysis demonstrated an increased net benefit of the model combining clinical variables and sTREM-1 over a clinical variable model alone, most notably in the decrease in false-positive prediction of death (see Additional files 6 and 8).

**Table 4:** Discrimination of mortality by LASSO-selected biomarkers

Model	AUC	95% CI	*p* value^a^
IL-8+Ang-2+sTREM-1	0.83	0.79–0.87	Ref
IL-8	0.77	0.73–0.81	0.002
Ang-2	0.77	0.73–0.82	0.0002
sTREM-1	0.81	0.77–0.85	0.07

^a^Test of equality between AUC of listed models and LASSO-selected IL-8+Ang-2+sTREM-1 model

**Table 5:** Clinical variable and sTREM-1 models of mortality prediction

Model	AUC	95% CI	*p* value^b^	*p* value^c^
Clinical variables^a^	0.79	0.74–0.84	Ref	–
sTREM-1	0.81	0.77–0.85	0.43	Ref
Clinical variables + sTREM-1	0.83	0.79–0.87	0.004	0.05

^a^Clinical variables model includes age, sex, Charlson Comorbidity Index, transfer status, and modified SOFA score

^b^Test of equality between AUC of listed models and the clinical variable model

^c^Test of equality between AUC of listed models and sTREM-1 model

### sTREM-1 increases with clinical severity of illness determined by modified SOFA score

**Fig. 2:**
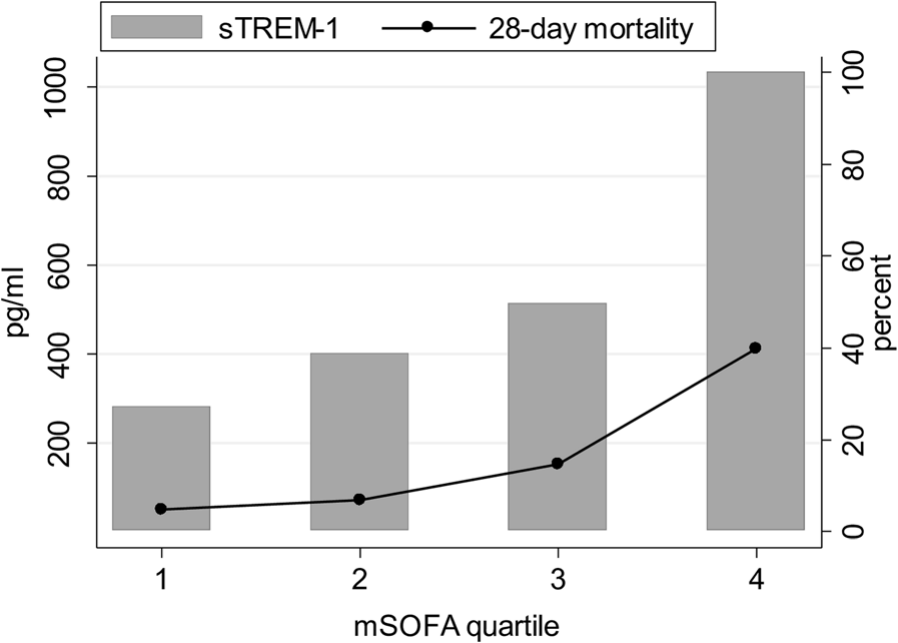
sTREM-1 and death at 28 days by modified SOFA score. Concentrations of sTREM-1 (pg/ml) and proportion of non-survivors at 28-days (%) are shown by quartile of modified SOFA score

As sTREM-1 alone and a clinical variable model including a modified SOFA score were comparable classifiers for the discrimination of death, we assessed the relationship between sTREM-1 levels and modified SOFA scores. sTREM-1 concentrations were positively correlated with quartile of modified SOFA score (Fig. 2, *r* = 0.53, *p* < 0.001).

## Discussion

In this study, we validated the performance of several biomarkers in predicting death from infection in a tropical, middle-income country. In a relatively large cohort of patients with infection admitted to a referral hospital either through the ED or by transfer from an outside facility, a single measurement of sTREM-1 within 24 h of admission is similar to clinical illness score models in predicting 28-day mortality. Combining sTREM-1 with a clinical illness score model further improves discrimination.

The global burden of sepsis is highest—but the fewest studies of sepsis are performed—in resource-limited settings [1]. Due to significant differences in etiologies, host factors, and clinical management of sepsis, it is essential to perform clinical studies in these at-risk populations rather than to extrapolate from high-income settings. Several features differentiate our study population and therefore expand the validity of the selected biomarkers for the prediction of outcome in sepsis. First, in comparison with most prior studies, the etiologies of infection at the study hospital and surrounding regions are distinct and heterogeneous: *Staphylococcus aureus*, *Escherichia coli*, *Burkholderia pseudomallei*, leptospirosis, scrub and murine typhus, dengue, and malaria are all described [3, 6, 7, 11]. Second, in contrast to many high-resource settings, due to intensive care unit bed availability at the study hospital, management of many patients with sepsis—even with respiratory failure or shock—frequently occurs on the general medical wards [8]. Our results provide important validation of the performance of sTREM-1 in mortality prediction in these populations.

Besides sTREM-1, all the other biomarkers we studied, with the exception of Ang-1, were strongly associated with 28-day mortality; moreover, discrimination of mortality by IL-8 and Ang-2 as measured by the AUC was 0.77 for each analyte. Others have reported that biomarkers of endothelial quiescence vs activation, such as Ang-1, Ang-2, and the ratio of Ang-2:Ang-1, are strong predictors of mortality in sepsis [16]. In Thai adults with malaria, Ang-1 levels decreased and Ang-2 levels increased with higher severity of illness [33]. As previously noted, similar patterns, as well as elevation in the Ang-2:Ang-1 ratio, have been reported in sepsis, though not in Southeast Asia or resource-limited settings [20]. Non-specific markers of inflammation, including IL-8 and sTNFR-1, also predict sepsis-related mortality [14]. Therefore, Ang-2, IL-8, and sTNFR-1 may have additional value in sepsis outcome prediction in a resource-limited setting and deserve further study.

Triggering receptor expressed on myeloid cells 1 (TREM-1) is a transmembrane receptor typically found on monocytes and neutrophils [34]. Activation of TREM-1 may play a role in innate inflammatory responses by modulating the secretion of pro-inflammatory cytokines such as IL-8 and TNF-α. Importantly, the activating ligand remains somewhat unclear, though during inflammation TREM-1 is released in a soluble form as sTREM-1 [35]. As sTREM-1 levels are elevated in non-infectious inflammatory states, results of its performance for diagnosis of sepsis have been mixed [15, 36, 37]. Our findings do suggest that a biomarker of innate immune activation has strong discrimination of mortality in a LMIC setting and may represent a distinct pathway compared to the other biomarkers we evaluated. Recently, in a cohort of outpatient, febrile adults in Tanzania, sTREM-1 enhanced clinical prediction of mortality [38]. Similarly, in our study, sTREM-1 was not only comparable to clinical variables in predicting mortality but enhanced mortality prediction when combined with clinical variables. However, of note, substantial differences exist between these studies, including patient setting, geographic location, rates of HIV infection, infectious etiologies, sample size, clinical scoring system, and method of biomarker measurement. Even considering these differences, our results provide additional evidence supporting the use of sTREM-1 to augment sepsis mortality prediction in tropical, resource-limited settings, particularly in Southeast Asia.

Organ failure assessment using SOFA scores may be challenging to determine in lower-resourced areas due to the lack of available data, prompting the development of simpler clinical prediction models [10, 39, 40]. Importantly, clinical parameters have associated costs, primarily related to healthcare infrastructure [41]. However, a potential advantage of a predictive biomarker is obviating the need to calculate clinical scores of any type and, when considered within a health system, may be cost-effective. Alternatively, in settings where clinical scores can be readily calculated, a biomarker may further augment mortality prediction. In our study, a single biomarker, sTREM-1, had similar outcome prediction as a clinical model including a modified SOFA score and may be a useful substitute in remote settings where certain clinical and laboratory data are difficult to ascertain.

Certain physiologic biomarkers, such as an elevated lactate, predict mortality in LMIC settings [42]. However, the early use of sepsis biomarkers to predict clinical trajectory may inform triage and resuscitation approaches in resource-limited settings [43]. Therefore, significant attention has focused on the utility of the inflammatory markers procalcitonin and C-reactive protein, both of which are widely available but have variable performance in Southeast Asia for infection prognosis [12, 13]. Assays for our analyzed biomarkers, including sTREM-1, are not readily available in clinical laboratories. However, our results may inform the future development of a simple point-of-care assay [44, 45]. Such an assay could help guide clinical decision-making about management and referral. Further studies will need to assess whether markers of innate immune activation, like sTREM-1, have superior sepsis-related outcome prediction compared to more general inflammation markers such as procalcitonin and C-reactive protein.

Our study has several strengths. To our knowledge, this is the largest study of a population of hospitalized patients to evaluate the performance of sTREM-1 in predicting outcomes from infection and the first to examine patients in Southeast Asia. We included in our analysis patients presenting primarily to the study hospital ED as well as patients referred from a variety of surrounding hospitals in the region, increasing the external validity of our results. Sample processing, tracking, and assaying were carefully implemented. There were minimal missing data and loss to follow-up in this cohort. Finally, several methods were used to validate the performance of sTREM-1.

Our study has several potential limitations, including those previously noted. While detailed clinical history as well as timely blood tests was obtained, resource limitations precluded certain tests, including arterial blood gases. Similarly, dosages of vasopressor or inotropic medications were not available. These factors limited our ability to completely calculate SOFA criteria. Although we used a modified SOFA score, this potentially limits the broader applicability of our findings. The study was performed at a single public provincial hospital, although the referral system in Thailand facilitates the expeditious transfer of sick patients from over 60 surrounding hospitals to this center, resulting in a sizeable catchment area [11]. For patients transferred from another facility, the extent of care received at the transferring facilities could not be assessed. Similarly, patients admitted to non-medical services, such as surgical wards, were not enrolled and may have different characteristics than our cohort. Our biomarker analysis plan was designed to select a parsimonious model and minimize bias. Therefore, we only analyzed the mortality discrimination of LASSO-selected biomarkers. However, other biomarkers, including sTNFR-1, may have similar discrimination to those analyzed. Finally, our study occurred in a specific region of Thailand, and generalization of these results to other locations with variable genetic, economic, and infectious characteristics may be limited.

## Conclusions

In summary, in a large observational study of adults in northeast Thailand hospitalized with an infection, multiple biomarkers of endothelial activation/dysfunction and innate immune activation, assayed once within 24 h of admission, are predictive of death. sTREM-1 has a similar mortality prediction compared to a clinical variable model that includes a modified SOFA score. sTREM-1 can further augment the clinical prediction of mortality when combined with clinical variables. These findings add new evidence to support the utility of blood biomarkers in predicting outcomes from infection and sepsis of diverse etiologies in tropical, resource-limited settings. With further study, such biomarkers may be helpful tools to guide clinical triage and resource allocation decisions for optimal patient management.

## Supplementary information

**Additional file 1.** Patient characteristics stratified by transfer status.

**Additional file 2.** Association of biomarkers with death stratified by transfer status.

**Additional file 3.** Association of Ang-2 with death stratified by transfer status.

**Additional file 4.** Adjustment and verification of sTREM-1 predictive models.

**Additional file 5.** Mortality prediction using models of clinical variables with or without sTREM-1.

**Additional file 6.** Net benefit of adding sTREM-1 to a model of clinical variables for mortality prediction.

**Additional file 7: Figure S1.** Biomarker selection using LASSO regression.

**Additional file 8: Figure S2.** Decision curve analysis.

## Data Availability

The dataset is available on reasonable request from the corresponding author (TEW).

## Abbreviations

Ang-1: Angiopoietin-1
Ang-2: Angiopoietin-2
AUC: Area under the receiver operating curve
CI: Confidence interval
ED: Emergency department
IDI: Integrated discrimination improvement analysis
IL-8: Interleukin-8
IQR: Interquartile range
LASSO: Least absolute shrinkage and selection operator
LMICs: Low- and middle-income countries
LR: Likelihood ratio
OR: Odds ratio
PaO_2_: Partial pressure of oxygen in arterial blood
SOFA: Sequential Organ Failure Assessment
sTNFR-1: Soluble tumor necrosis factor receptor 1
sTREM-1: Soluble triggering receptor expressed by myeloid cells 1
TREM-1: Triggering receptor expressed on myeloid cells 1
