# Supplementary material for: sTREM-1 predicts mortality in hospitalized patients with infection in a tropical, middle-income country
Source: BMC Med. 2020 Jul 1;18:159. doi: 10.1186/s12916-020-01627-5 (PMC7329452; doi:10.1186/s12916-020-01627-5)
Supplement: Supplementary file 1 — Additional file 1. Patient characteristics stratified by transfer status. [file 12916_2020_1627_MOESM1_ESM.pdf]

Additional file 1: Patient characteristics stratified by transfer status

Characteristics	Not transferred (n=380)	Transferred (n=380)
Demographics		
Age in years, median (IQR)	59 (36-72)	60 (44-74)
Male sex, N (%)	179 (47)	222 (58)
Pre-existing conditions		
Charlson Comorbidity Index, median (IQR)	2 (0-4)	2 (1-4)
Diabetes, N (%)	79 (21)	78 (21)
Chronic liver disease, N (%)	7 (2)	11 (3)
Chronic kidney disease, N (%)	39 (10)	46 (12)
Chronic cardiovascular disease, N (%)	25 (7)	22 (6)
Chronic lung disease, N (%)	32 (8)	28 (7)
Cancer, N (%)	12 (3)	5 (1)
HIV, N (%)	5 (1)	4 (1)
Modified SOFA score, median (IQR)	1 (0-3)	4 (3-7)
Subjects with modified SOFA score ≥2, N (%)	166 (44)	326 (86)
Died within 28 days, N (%)	40 (11)	70 (18)
