# Supplementary material for: sTREM-1 predicts mortality in hospitalized patients with infection in a tropical, middle-income country
Source: BMC Med. 2020 Jul 1;18:159. doi: 10.1186/s12916-020-01627-5 (PMC7329452; doi:10.1186/s12916-020-01627-5)
Supplement: Supplementary file 2 — Additional file 2. Association of biomarkers with death stratified by transfer status. [file 12916_2020_1627_MOESM2_ESM.pdf]

Additional file 2: Association of biomarkers with death stratified by transfer status

Biomarker[a]	Transfer	Unadjusted		Modified SOFA-adjusted[c]	
		OR (95% CI)	Interaction p value[b]	OR (95% CI)	Interaction p value[b]
IL-8	No	2.8 (2.0-3.9)	0.16	2.0 (1.3-3.1)	0.24
	Yes	2.0 (1.6-2.6)		1.8 (1.3-2.4)	
sTNFR-1	No	46.2 (14.1-151.1)	0.07	6.1 (1.5-25.5)	0.11
	Yes	11.5 (4.7-27.8)		4.1 (1.5-11.7)	
Ang-1	No	1.1 (0.6-2.0)	0.84	1.7 (0.7-4.0)	0.63
	Yes	1.2 (0.8-1.9)		1.1 (0.7-1.9)	
Ang-2	No	23.4 (9.2-59.7)	0.03	6.0 (1.9-18.9)	0.14
	Yes	6.6 (3.4-13.0)		4.2 (1.9-9.3)	
Ang-2:Ang-1	No	3.0 (1.8-4.8)	0.08	1.4 (0.7-2.7)	0.47
	Yes	1.7 (1.2-2.5)		1.4 (0.9-2.2)	
sTREM-1	No	56.5 (18.7-170.3)	0.17	11.1 (2.7-45.1)	0.28
	Yes	20.5 (7.9-53.0)		9.3 (3.2-27.0)	

[a] Biomarkers were log_{10} transformed before regression; the ORs (95% CI) of death reported are for each biomarker after stratification by transfer status

[b] p values for interaction of transfer status/set and biomarker were generated by a logistic regression model of death including biomarker, transfer status, and an interaction term.

[c] Models were adjusted for age, sex, Charlson Comorbidity Index, and modified SOFA score
