# Supplementary material for: sTREM-1 predicts mortality in hospitalized patients with infection in a tropical, middle-income country
Source: BMC Med. 2020 Jul 1;18:159. doi: 10.1186/s12916-020-01627-5 (PMC7329452; doi:10.1186/s12916-020-01627-5)
Supplement: Supplementary file 3 — Additional file 3. Association of Ang-2 with death stratified by transfer status. [file 12916_2020_1627_MOESM3_ESM.pdf]

Additional file 3: Association of Ang-2 with death stratified by transfer status

**Biomarker**[a]	**Transfer**	**Unadjusted**		**Modified SOFA-adjusted**[b]	
		**OR (95% CI)**	**p value**	**OR (95% CI)**	**p value**
Ang-2	No	23.4 (9.2-59.7)	<0.001	6.0 (1.9-18.9)	<0.001
	Yes	6.6 (3.4-13.0)	<0.001	4.2 (1.9-9.3)	<0.001

[a] Biomarker was log_{10} transformed before regression
[b] Models were adjusted for age, sex, Charlson Comorbidity Index, and modified SOFA score
