# Supplementary material for: sTREM-1 predicts mortality in hospitalized patients with infection in a tropical, middle-income country
Source: BMC Med. 2020 Jul 1;18:159. doi: 10.1186/s12916-020-01627-5 (PMC7329452; doi:10.1186/s12916-020-01627-5)
Supplement: Supplementary file 4 — Additional file 4. Adjustment and verification of sTREM-1 predictive models. [file 12916_2020_1627_MOESM4_ESM.pdf]

Additional file 4: Adjustment and verification of sTREM-1 predictive models

Model	AUC (95% CI)	Optimism-adjusted AUC (95% CI)[a]	IDI[b]
Clinical variables	0.79 (0.74-0.84)	0.79 (0.74-0.83)	ref
sTREM-1	0.81 (0.77-0.85)	0.81 (0.76-0.85)	-
Clinical variables + sTREM-1	0.83 (0.79-0.87)	0.83 (0.79-0.87)	0.05 ± 0.01 P<0.001

[a] Optimism adjustment: bias correction based on 1000 x internal replication by bootstrapping

[b] IDI: Integrated discrimination improvement; values represent model improvement estimate of a clinical variable model with sTREM-1 over the clinical variable model alone ± standard error
