# Supplementary material for: sTREM-1 predicts mortality in hospitalized patients with infection in a tropical, middle-income country
Source: BMC Med. 2020 Jul 1;18:159. doi: 10.1186/s12916-020-01627-5 (PMC7329452; doi:10.1186/s12916-020-01627-5)
Supplement: Supplementary file 5 — Additional file 5. Mortality prediction using models of clinical variables with or without sTREM-1. [file 12916_2020_1627_MOESM5_ESM.pdf]

Additional file 5: Mortality prediction using models of clinical variables with or without sTREM-1

Model	Variable	OR	95% CI	p value
**Clinical variables**	Age	1.01	0.99-1.03	0.49
	Female sex	0.92	0.57-1.46	0.71
	Charlson Comorbidity Index	1.20	1.02-1.42	0.03
	Modified SOFA score	1.38	1.28-1.48	<0.001
**Clinical variables + sTREM-1[a]**	Age	1.01	0.98-1.03	0.60
	Female sex	0.83	0.51-1.34	0.44
	Charlson Comorbidity Index	1.10	0.92-1.31	0.28
	Modified SOFA score	1.22	1.13-1.33	<0.001
	sTREM-1	11.1	4.90-25.16	<0.001

[a]Model containing clinical variables and $\log_{10}$ sTREM-1 differed significantly by likelihood ratio test ($P=1.6 \times 10^{-9}$) compared to the clinical variable model.
