# Supplementary material for: sTREM-1 predicts mortality in hospitalized patients with infection in a tropical, middle-income country
Source: BMC Med. 2020 Jul 1;18:159. doi: 10.1186/s12916-020-01627-5 (PMC7329452; doi:10.1186/s12916-020-01627-5)
Supplement: Supplementary file 6 — Additional file 6. Net benefit of adding sTREM-1 to a model of clinical variables for mortality prediction. [file 12916_2020_1627_MOESM6_ESM.pdf]

Additional file 6: Net benefit of adding sTREM-1 to a model of clinical variables for mortality prediction

Probability of death (%)	Model net benefit[a]				Advantage of adding sTREM-1 to Clinical Variable model	
	Predict all die	Clin. Var.	Clin. Var. + sTREM-1	Advantage of adding sTREM-1	Increase in number correctly predicted to die per 100 patients[b]	Reduction in number incorrectly predicted to die per 100 patients[c]
5	0.100	0.107	0.109	0.003	0.2	4.9
10	0.050	0.077	0.089	0.012	1.0	11.2
15	-0.006	0.065	0.076	0.011	0.5	6.1
20	-0.069	0.042	0.058	0.016	1.4	6.3
25	-0.141	0.042	0.043	0.001	-0.4	0.4
30	-0.222	0.028	0.034	0.005	-0.4	1.2

[a] Model benefit derived from ((true-positive count/N)-(false-positive count/N)(probability of death/1- probability of death))

[b] Increase in number correctly predicted to die per 100 patients for each model calculated as: (net benefit of the model – net benefit of assuming all patients will survive 28 days)/(probability of death/(1-probability of death))*100). This number is net of false positive predictions of death.

[c] Reduction in number incorrectly predicted to die per 100 patients for each model calculated as: (net benefit of the model – net benefit of assuming all patients will die in 28 days)/(probability of death/(1-probability of death))*100). This number is net of false negative predictions of death.
