# Supplementary material for: sTREM-1 predicts mortality in hospitalized patients with infection in a tropical, middle-income country
Source: BMC Med. 2020 Jul 1;18:159. doi: 10.1186/s12916-020-01627-5 (PMC7329452; doi:10.1186/s12916-020-01627-5)
Supplement: Supplementary file 7 — Additional file 7: Figure S1. Biomarker selection using LASSO regression. [file 12916_2020_1627_MOESM7_ESM.pdf]

Additional file 7: Figure S1. Biomarker selection using LASSO regression

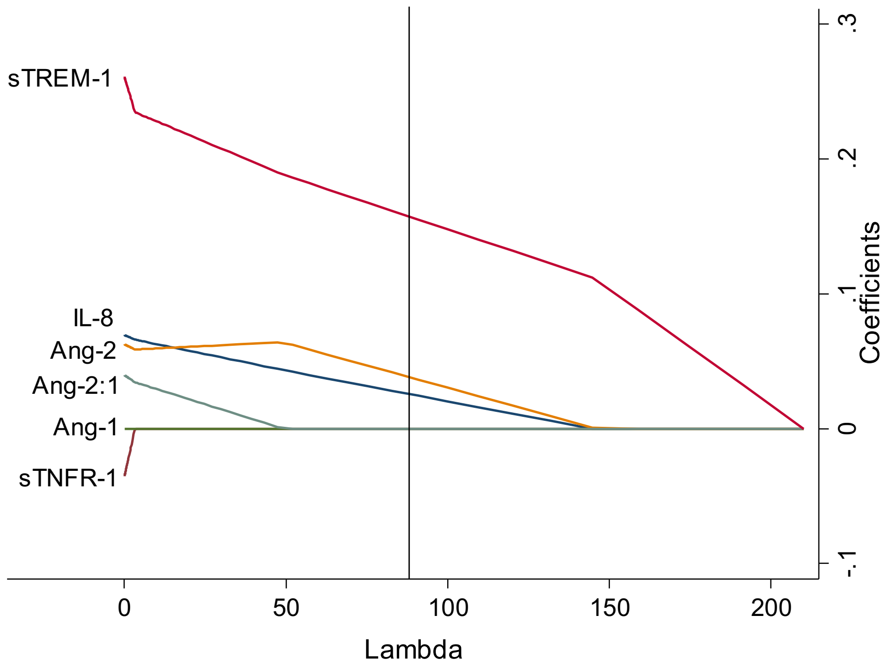

LASSO coefficient profiles of the six analyzed variables are depicted. The vertical line notes the largest lambda (91) for which the mean squared prediction error (MSPE) is within one standard error of the minimal MSPE in 10-fold cross validation. The biomarkers to the right of the vertical line (sTREM-1, IL-8 and Ang-2) were selected by LASSO.
