# Supplementary material for: sTREM-1 predicts mortality in hospitalized patients with infection in a tropical, middle-income country
Source: BMC Med. 2020 Jul 1;18:159. doi: 10.1186/s12916-020-01627-5 (PMC7329452; doi:10.1186/s12916-020-01627-5)
Supplement: Supplementary file 8 — Additional file 8: Figure S2. Decision curve analysis. [file 12916_2020_1627_MOESM8_ESM.pdf]

**Additional file 8: Figure S2. Decision curve analysis.**

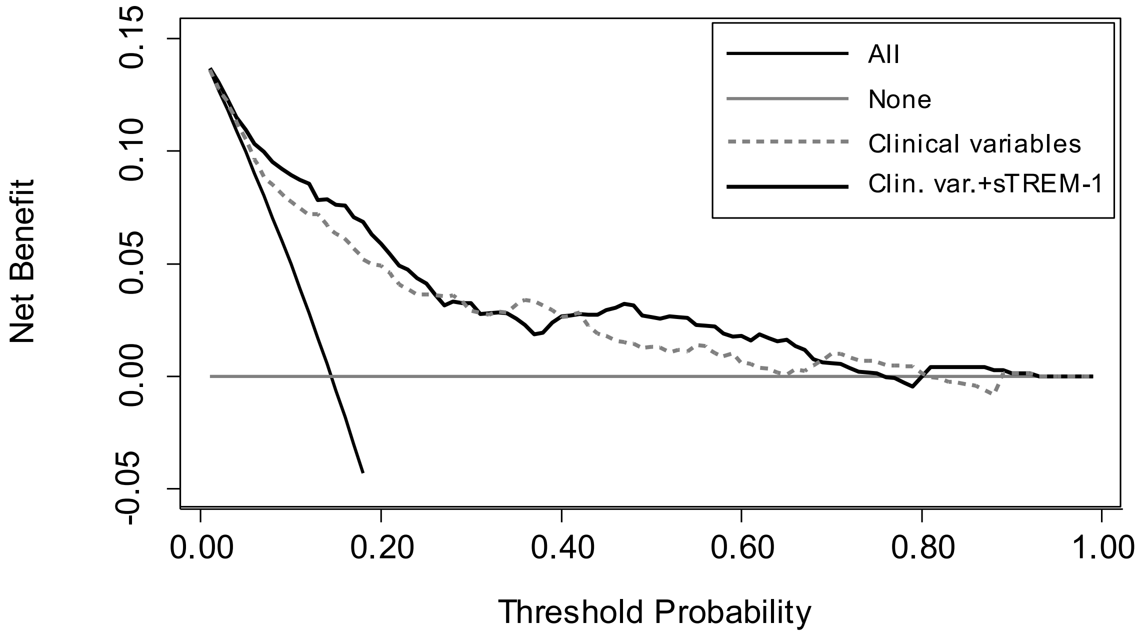


Decision curves for 28-day mortality prediction. The net benefit of the model is presented on the y-axis and the probability of death in 28 days is represented on the x-axis. The net benefit of the clinical variable model is shown with a dotted gray line and the net benefit of a model combining clinical variables and sTREM-1 is shown with a thick black line. The diagonal thin black line represents the assumption that all subjects will die within 28 days and the solid gray line represents the assumption that no subjects will die within 28 days.
